# Does flooding effect the apparent survival and body condition of a ground foraging migrant passerine?

**DOI:** 10.1371/journal.pone.0175179

**Published:** 2017-04-10

**Authors:** Bryan M. Reiley, Thomas J. Benson, Jeremy Everitts, James C. Bednarz

**Affiliations:** 1 Department of Biological Sciences, Arkansas State University, Jonesboro, Arkansas, United States of America; 2 Illinois Natural History Survey, Prairie Research Institute, University of Illinois, Champaign, Illinois, United States of America; 3 Department of Biological Sciences, University of North Texas, Denton, Texas, United States of America; Sichuan University, CHINA

## Abstract

Natural disturbances play a fundamental role in maintaining habitat and landscape heterogeneity; however, these events can also have negative effects on some species. While we know that disturbances can reduce habitat quality for many species, leading to diminished populations and altered community structure, the effect of these events on individuals that continue to occupy affected areas remains unknown. We focused on understanding the impact of flood-mediated reduction of habitat quality on Swainson’s Warblers (*Limnothlypis swainsonii*). In 2008, a catastrophic flood event occurred on the Mississippi River and its tributaries, severely affecting one of two locations where we had studied territorial males since 2004. To determine the impact of flooding on this species, we evaluated how body condition and apparent survival of males differed between locations and in pre-flood (2004–2007) and post-flood (2008–2010) periods. Body condition did not differ between locations after the flood, suggesting that flooding did not cause food limitation for this obligate ground forager. Apparent survival in the post-flood period was lower at both locations and led to near population extirpation at the heavily flood-impacted site. Overall, this study demonstrates the vulnerability of species to extreme hydrological events, an increasing threat due to climate change. Future research should focus on identifying species that are vulnerable to these events and determining appropriate conservation strategies. Conservation for the Swainson’s Warbler should focus on identifying and conserving the highest elevation, least flood prone areas within bottomland hardwood forests and managing those areas for thick understory vegetation.

## Introduction

Natural disturbances play a fundamental role in creating habitat and landscape heterogeneity that benefits various taxa [1]. For example, flooding is known to create and maintain diverse environments within riparian ecosystems by influencing plant communities [2] and regulating nutrient processing [3]. As a result, floodplains are highly productive and diverse ecosystems [4]. Yet, floods can differ greatly in prevalence, timing, extent, and intensity [5–7] and, despite the benefits; extreme hydrological events can have negative proximate impacts to many taxa [8–10]. This is particularly important for species of concern, where extreme flood events can threaten conservation efforts. Ultimately, understanding how species respond to floods is especially relevant given expected increases in climatic variability due to global climate change [11], which may lead to more frequent extreme hydrological events [12,13].

For birds, responses to extreme flooding can vary depending on the spatio-temporal extent of the event and their ecology. Changes in habitat as a result of prolonged flows have been correlated with subsequent changes in density, occupancy, and community structure [14–16]. Within the Mississippi Alluvial Valley, flooding has been documented to alter understory vegetation in floodplain forests. Specifically, understory dependent and ground foraging bird species appear to be especially sensitive to extreme flood events [14, 15, 17, 18].

Beyond evaluating changes in habitat use by populations and communities of birds due to flood-induced habitat alteration, studies have shown these events can have consequences to individuals. For example, flood events have caused direct affects to birds by causing nest failures [19–21] however in at least one case flooding increased nest success due to predator exclusion [22]. Additionally, flooding can have indirect effects on birds by affecting the types and amount of prey items [23], which can affect adult body condition and apparent survival [24] both known to be important for population trajectories [25,26]. As a consequence, understanding how flooding affects these demographic parameters is important for species of conservation concern that are affected by extreme hydrological events.

One such species is the Swainson’s Warbler (*Limnothlypis swainsonii*). This warbler species breeds in the southeastern United States, winters in the Caribbean Basin, and inhabits areas with dense understory vegetation [27]. Swainson’s Warblers are uncommon and local throughout their breeding range, and are ranked among the highest-priority species for conservation [28]. Swainson’s Warblers forage for arthropods in terrestrial dead leaf litter, primarily by flipping dead leaves and poking their bills into litter on the ground [29–32]. Primary habitat for this species is bottomland hardwood forests of the southeastern US [27]. Within bottomland hardwood forests, this species has shown elevational preferences by selecting to breed in only the least flood prone areas [33]. However, field studies have documented abandonment of previously occupied territories as a result of extreme flooding events [34, 35, 16, 17]. Unfortunately, the highest elevation areas of bottomland hardwood forests have been mostly converted to agriculture [36] and remaining habitat has been subjected to increased depth and duration flood events as a result of existing flood control levees [34,37,16]. Thus, understanding the demographic response of Swainson’s Warblers to flooding in these habitats has important conservation implications for populations of this and other species using bottomland hardwood forests.

To study demographic responses of Swainson’s Warbler to flooding intensity, we investigated the influence of flooding on body condition and male apparent survival. We examined these responses in a system where we have studied marked populations of males for both 4 years pre-flood and 3 years’ post-flood, providing a unique opportunity to evaluate how flooding affects habitat quality for this species of conservation concern. Previous research [18] found that territory abandonment of males after prolonged inundation lasting more than 1 month was associated with reduced leaf litter depth and cover, arthropod abundance of known Swainson’s Warbler prey, and shrub densities. Yet, it is not known how these changes affect birds that continue to occupy territories during and after flood events. We assume a reduction in these habitat variables and reduced arthropod abundance likely would diminish habitat quality and lead to diminished habitat quality for an understory dependent ground foraging species such as the Swainson’s Warbler. To assess if the reductions of these variables subsequently reduced habitat quality for individuals continuing to occupy territories after the flood, we monitored and assessed indirect measures of habitat quality including body condition and apparent survival [38]. We attempted to accomplish this by investigating differences and temporal changes in body condition and apparent survival at two locations with different flooding influences. Previous research suggested that body condition increased throughout the breeding season [39] and so we predicted that if habitat quality were reduced, body condition would remain stable or decrease throughout the breeding season at the heavily flooded site. In addition, we expected body condition and apparent survival of birds at the heavily flooded site would be lower as a result of reduced habitat quality due to the extended period of inundation compared to estimates at the site with marginal flooding in the post-flood time period.

## Materials and methods

### Study area

During the early spring of 2008, record rainfall fell in the Midwestern U.S., leading to above average flows in the rivers in the Upper Mississippi river drainage. The above average flows caused catastrophic flooding along many Midwestern Rivers and their floodplains, flooding that was exacerbated by the extensive levee system that is in place to control flooding [40]. This extensive flooding affected two study sites where we have monitored territorial males since 2004 at the White River National Wildlife Refuge (WR) and Saint Francis National Forest (SF) [41–44]. Though both sites were affected by the flooding, WR was completely inundated for 1 month during the breeding season and SF only experienced minor flooding in < 10% of the previously occupied territories.

From late April to early August 2004 through 2007, Benson et al. [39] used passive and song-playback surveys to locate, capture, and color band territorial males at WR and SF. During the spring and summers of 2008 through 2010 we resurveyed all previously occupied territories and any potential surrounding habitat at both of these field sites.

SF is a 9150 ha mix of mostly upland forest and small patches of bottomland forest located at the southern tip of Crowley’s Ridge. This site is located in Lee and Phillips counties in southeast Arkansas and bordered to the east by the St. Francis and Mississippi rivers.

WR, also located in Southeast Arkansas and 48 miles southwest of SF, is one of the largest contiguous tracts of remaining bottomland hardwoods in the southeastern U.S. Our study site is a relatively high elevation area near Alligator Lake in the southern portion of the refuge. Occupied habitat at WR is slightly higher in elevation, and therefore, less prone to flooding than much of the rest of the refuge.

### Survey technique

During the spring and summer of 2008 through 2010 we systematically resurveyed all previously occupied territories during 2004 through 2007. Surveys were conducted three times per year in 2008 and 2009 and two times per year in 2010. We attempted to re-sight all returning males using passive surveys for 10 minutes and song playbacks for 10 minutes in each previously occupied territory and all k potential habitat within the study areas. All un-banded males were captured using targeted mist netting with song playbacks and fitted with a metal U.S. Geological Survey band as well as a unique combination of three color bands (U.S.G. S. Master bander permit # 21426). Once a bird was captured and banded, we measured the length of the bill from the anterior edge of the nares to the tip, the left and right tarsi (middle of midtarsal joint to the distal end of tarsometatarsus), the left and right unflattened wing chord, and the standard tail length, to the nearest 0.5 mm. We also recorded the mass of the bird using Pesola and digital scales to the nearest 0.5 g.

### Analyses

We generated body condition index scores for captured males by first using principal component analysis to generate one variable (PC1) from our six correlated linear measurements and then used residuals from a linear regression between this linear size variable and body mass as an index of condition [45,46]. Birds with positive scores were considered in good condition and those with negative scores were in relatively poor condition.

Using body mass adjusted to body size, we evaluated temporal effects of pre-flood and post-flood time periods, day of year, location, and additive and interactive combinations of these variables. Body condition data from 2004 was excluded from this analysis due to differences in measurement techniques. Models were chosen based on previous literature i.e., [34] and what we felt was biologically relevant to Swainson’s Warblers after a large scale flood event (Table 1). We fit candidate models using general linear mixed models (SAS PROC GLIMMIX; [47]) and evaluated results from these models using Akaike’s Information Criterion adjusted for small sample size AIC_*c*_, [48] and relative model weights based on AIC_*c*_ rankings. Additionally, we used the sum of model weights across models including particular variables as indicators of variable importance. To account for non-independence of resampled individuals, we treated bird identification as a random effect.

**Table 1:** Results from best-fitting temporal and location models used to predict body condition of Swainson's Warblers (*n* = 278)at White River National Wildlife Refuge and Saint Francis National Forest, eastern Arkansas 2005–2010.

Model	K^a^	ΔAIC_*c*_^b^	w_*i*_
flood × day of year	4	0	0.814
flood + day of year	3	3.09	0.174
flood + location	4	9.35	0.008
flood × location	4	10.91	0.003
flood	2	13.57	0.001
day of year	2	23.4	0.000
null (intercept only)	1	35.95	0.000

^a^ number of parameters.

^b^ AIC_*c*_ for the best ranked model was 686.44.

We estimated apparent survival using Cormack-Jolly-Seber methods [49] in program MARK [50]. We evaluated multiple hypotheses for variation in apparent survival and recapture probability at the WR and SF (Table 2) and ranked these models using Akaike’s Information Criterion adjusted for small sample sizes. Year effects between study sites for capture/recapture probability were not evaluated in models due to comparable efforts at each location over time. We fit models considering possible variation between locations and years (i.e. pre-flood and post-flood time periods), and constant apparent survival.

**Table 2:** Results from the top models used to estimate annual apparent survival (Phi) and resighting probabilities for male Swainson's Warblers (*n* = 254) at 2 locations in Arkansas, White River National Wildlife Refuge (WR) and St. Francis National Forest (SF), 2005–2010.

Model	K^a^	ΔAIC_*c*_^b^	AIC_*c*_^c^	w_*i*_^d^
Φ _(pre-flood WRandSF_^e^_≠ post-flood080910_^f^_WRandSF)_ *p*	3	0.00	652.34	0.36
Φ _(pre-flood WRandSF ≠ post-flood080910 WRandSF)_ *p*_(pre-floodWRandSF≠post-flood0809WRandSF_^g^_)_	2	0.29	652.63	0.31
Φ _(pre-flood WRandSF ≠ post-flood080910 WRandSF)_ *p*_(pre-flood WRandSF≠post-flood080910 WRandSF)_	3	1.79	654.14	0.15
Φ _(pre-flood WRandSF ≠ post-flood 080910WRandSF)_ *p*_(pre-flood WRandSF≠post-flood08 WRandSF_ ^h^_)_	4	1.86	654.21	0.14
Φ *p*	2	7.64	659.99	0.01

^a^ Number of Parameters.

^b^ Δ AIC_*c*_ = AIC_*c*i_—minAIC_*c*_.

^c^ AIC_*c*_ = -2log *L* + 2K + 2K(K + 1)/(n-K-1).

^d^ *w*_*i*_ = exp[-{Δ AIC_*c*i_ /2}]/Sum exp[-{Δ AIC_*c*i_/2}].

^e^Pre-flood (2004–2007) at both WR and SF.

^f^Post-flood (2008–2010) at both WR and SF.

^g^Post-flood (2008 and 2009 only) at both WR and SF.

^h^Post-flood (2008 only) at both WR and SF.

## Results

From 22 April to 16 July 2005–2010 we captured, banded, weighed, and measured 278 territorial males, 122 at WR (35 in 2005, 26 in 2006, 22 in 2007, 28 in 2008, 6 in 2009, and 5 in 2010), and 156 at SF (13 in 2005, 22 in 2006, 18 in 2007, 62 in 2008, 35 in 2009, and 6 in 2010; Table 3) to analyze body condition. The first principal component (PC1) of our PCA analysis explained 40% of the variation (eigenvalue = 2.39) of our linear measurements and all variables loaded positively (Table 4). There was a slight positive relationship between PC1 and mass (mass = 0.14 * PC1 + 15.67; *r*^2^ = 0.018). Mean condition estimates for WR and SF were −0.37 (n = 82) and −0.16 (*n* = 52) pre-flood, and −0.06 (*n* = 38) and 0.31 (*n* = 102) post-flood, respectively, four birds were excluded from analysis due to missing measurements.

**Table 3:** Mean, standard error, and range of values for morphological measurements of male Swainson's Warblers (*n* = 278) captured at White River National Wildlife Refuge and Saint Francis National Forest, 2005–2010.

Variable	Mean	SE	Range
Bill (mm)	11.36	0.04	10.0–13.70
Left leg (mm)	17.46	0.04	15.7–19.0
Right leg (mm)	17.50	0.04	15.0–19.0
Left wing (mm)	70.06	0.10	66.7–75.0
Right wing (mm)	70.19	0.10	66.0–75.0
Tail (mm)	47.47	0.17	30.0–53.0
Mass (g)	15.64	0.05	13.0–18.0

**Table 4:** Principal component loadings for 6 linear measurements collected from the Swainson's Warblers (*n* = 278) at White River National Wildlife Refuge and Saint Francis National Forest, 2005–2010.

	PC1	PC2
Eigenvalue	2.39	1.64
Percent Variance	0.40	0.27
Variable		
Bill	0.42	0.23
Left leg	0.67	0.63
Right leg	0.65	0.68
Left wing	0.71	-0.57
Right wing	0.70	-0.59
Tail	0.42	0.23

**Fig 1:**
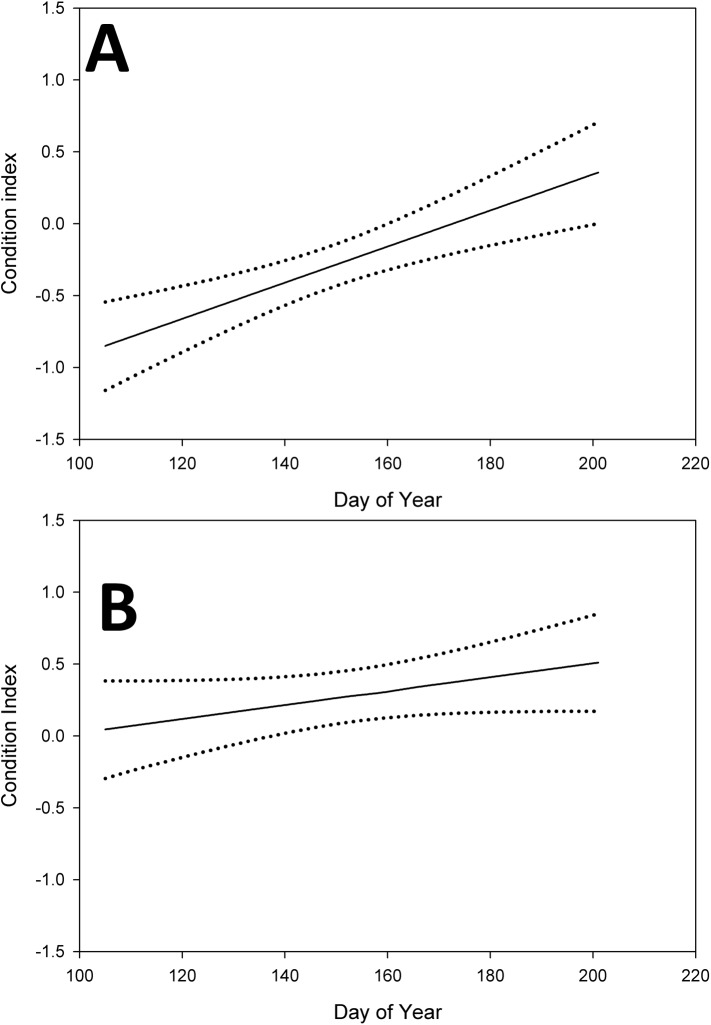
Model-averaged relationship between body condition index (with 95% confidence intervals) of male Swainson’s Warblers and day of year through the breeding season in (A) the pre-flood period (2005–2007) at White River National Wildlife Refuge and Saint Francis National Forest and (B) the post-flood period (2008–2010) at White River National Wildlife Refuge and Saint Francis National Forest.

There was strong evidence for a difference in condition between pre-flood and post-flood time periods (Σ*w*_i_ = 1.00) and an effect of day of year (Σ*w*_i_ = 0.98; Table 1) on condition, with the most support for a model considering the flood × day of year interaction. Models that included location effects were not well supported (Σ*w*_i_ = 0.01; Table 1). To investigate if there were differences in pre-flood and post-flood body condition and day of year we model averaged condition for both time periods at mean day of year. Pre-flood condition was -0.274 (95% C.I.: -0.420, -0.128) and post-flood condition was 0.201 (95% C.I.: 0.057, 0.343). Body condition increased throughout the breeding season at both locations in both pre-flood (Fig 1A) and post-flood time periods (Fig 1B), although the slope was greater during the pre-flood period (0.013) than the post-flood period (0.005).

To estimate apparent survival for territorial males, we resighted 254 individuals: 119 at WR (21 in 2004, 31 in 2005, 22 in 2006, 20 in 2007, 18 in 2008, 7 in 2009) and 135 at SF (6 in 2004, 11 in 2005, 19 in 2006, 17 in 2007, 60 in 2008, and 22 in 2009). Four models fit the data better than the constant-survival model with models with flood period effects at both locations accounting for 96% of the AIC_c_ weight (Table 2). Three models that included flood period effects on both apparent survival and resight/recapture probability fit better than the constant model and accounted for 60% of the AIC_c_ weight (Table 2). Contrary to expectation, no models with location effects ranked higher than the null model, indicating similar trends in apparent survival at both study sites. The top model indicated lower apparent survival for the post-flood period (Table 5).

**Table 5:** Model averaged estimates for adult apparent survival (*φ* with 95% confidence intervals) in pre-flood years (2004–2007) and post-flood years (2008–2010) for male Swainson's Warblers (*n* = 254) at White River National Wildlife Refuge and Saint Francis National Forest.

Year	*φ*	95% CI
Pre-flood	0.60	0.52–0.68
Post-flood	0.44	0.37–0.50

Resight/recapture probability for the top model was 0.84 (SE = 0.04). The second-ranked model indicated resight/recapture probability differing between flooding periods, pre-flood 0.80 (SE = 0.05) and post-flood 0.89 (SE = 0.05).

## Discussion

Our body condition analysis indicated that post-flood body condition increased throughout the breeding seasons at both study sites (Fig 1B), which was contrary to our expectations though it increased more steeply in the pre-flood period.

Additionally, though average post-flood body condition was lower at WR than SF, but models including location effects were not supported. Importantly, the best-supported model indicated an interaction between flood period and date such that post-flood body condition was greater than pre-flood at the beginning of the breeding season at both locations. However, body condition toward the end of the breeding season was similar during both periods, suggesting that habitat quality was not driving the differences we observed. Based on this evidence, we suggest that during flood events and subsequently, food resources did not limit male body condition in this species during the breeding season.

Our results indicate that something other than habitat quality on the breeding grounds could be influencing body condition of territorial males, such as conditions during the non-breeding season. There has been considerable study on the idea that bird populations are limited during the winter period e.g. [51, 52]. Specifically, food availability may be a limiting factor during this season and may lead to carry over effects [53]. Indeed, rainfall during the non-breeding season is a known driver of arthropod populations on the wintering grounds of warbler species [53] and may influence overall condition of warbler species during the non-breeding season[54,52]. For Swainson’s Warblers, whose wintering grounds include the Caribbean, Mexico, and Central America, increased winter rainfall patterns are influenced by Southern Oscillation weather patterns [55], commonly referred to as La Niña events. Both 2008 and 2009 were La Niña years in the Caribbean basin, Mexico, and Central America [56], which may have led to increased food availability on the wintering grounds potentially explaining why we observed greater average body condition during the post-flood period at the beginning of the breeding season and why both of our study sites had similar body condition trends even though WR was severely flooded.

Contrary to our expectations, apparent survival at WR was not lower than SF during the post-flood period. The best-supported model indicated reduced apparent survival at both locations in all years post-flood. These findings indicate both sites are exhibiting similar declines in apparent survival. Whether the differences in apparent survival represent actual survival or permanent emigration is unclear. At WR, the large-scale flood event could have caused direct mortalities as documented in other taxa [57,58]. However, observations of birds occupying territories and feeding on novel substrates during flooding events [16] suggest birds can survive short-term flooding. We suggest that birds arriving on the flooded breeding grounds dispersed to other drier habitats in the year of the flood and thereafter. Short-term flooding induced dispersal has been documented in various taxa including insects [59, 9, 60] and mammals [57]. For Swainson’s Warblers flooding was observed to cause short-distance dispersal during the year of the flood [16]. Meanley [61] found that flooding caused four previously occupied territories to become unoccupied and Klaus [35] found flooding related abandonment of eight territories. In addition to emigration directly related to inundated territories subsequent changes in habitat probably further discourage birds from settling there after flooding events. Benson and Bednarz [17] documented that flooding caused declines in shrub cover, litter depth, and litter cover led to reduced occupancy of territorial males in Arkansas. Likewise, we found reduced occupancy to be associated with declines in litter cover and understory stems [18] which are known to affect food resources and nest site selection for this species [44,17]. Though occupancy was reduced post-flood, at WR some birds chose to continue to attempt to breed in these habitats [16]. For these birds, reduced understory density might have led to lower nest success [44] and therefore dispersal as a result [62,63] leading to the significant drops in occupancy at WR in the second and third years post-flood [18]. Subsequent surveys at this site in 2014 found only two territories occupied and so it appears that this flooding event lead many birds to emigrate due to reduced habitat quality for birds at this site [18] leading to localized extirpation of this population (B. Reiley pers. obs).

Initially we hypothesized that similar factors could be influencing the apparent survival of territorial males at SF. Recent anthropogenic habitat alterations might have reduced the habitat quality for this species. During the winters of 2007 and 2009 the United States Forest Service carried out prescribed burns to reduce understory fuels in some areas previously occupied by territorial males [64]. We speculated that the prescribed burns instituted at SF might have led to increased emigration due to lower habitat quality and therefore reduced apparent survival of individuals continuing to occupy these areas; however, occupancy analysis suggested that burned territories had the same probability of being occupied as unburned territories in subsequent years, often by the same occupant as the previous year [64]. So why did we see lower apparent survival at the unflooded site? We suspect that the large number of males (60) occupying territories at SF in 2008, which was significantly more than the average number of territories (16) occupied in all other years, was driving the reduced apparent survival we observed at this site during the post-flood period. We are uncertain why there was a sudden increase in the number of territorial males in 2008, but only a small number of these males returned in subsequent years, 22 and 10 in 2009 and 2010, respectively. Reduced returns of these birds may have resulted from phenomena associated with the wintering grounds (e.g., habitat loss, drought) or migration (e.g., hurricanes). Alternatively, we propose that in 2008 regional scale flooding within the Mississippi Alluvial Valley may have displaced many populations of this species leading to the increased number of Swainson’s Warblers at SF. If so, many of these birds may have been occupying relatively lower quality territories in 2008 and so birds may have permanently emigrated out of those territories in subsequent years, leading to the lower apparent survivorship we observed.

While migratory species that occupy flood-prone areas have evolved strategies to emigrate and find alternative habitats, we found that extreme flow events can be locally detrimental to these species leading to lower apparent survival and local extirpation. While this study focused on one species, our results demonstrate the vulnerability of floodplain dependent species of concern to extreme flooding events which are an increasing threat due to climate change [11]. Future conservation strategies for these species will depend on identifying susceptible priority species and the habitats they occupy to identify locations for targeted conservation efforts. This could be efficiently accomplished through the use of broad scale species habitat modeling such as the vulnerability index suggested by Royan et al. [65]. Specifically, for Swainson’s Warblers, managers should identify and preserve the least flood-prone areas within large bottomland hardwood forest tracts [33,18]. Furthermore, managers should attempt to maintain thick understory vegetation in areas that reach late successional stages. Uneven aged forest management including group selection cuts combined with regular thinning like those suggested by [66] would accomplish these goals and ensure that thick understory vegetation would have the appropriate light to regenerate in the event of flooding.
