# Correction: Does flooding effect the apparent survival and body condition of a ground foraging migrant passerine?

**DOI:** 10.1371/journal.pone.0181493

**Published:** 2017-07-10

**Authors:** Bryan M. Reiley, Thomas J. Benson, Jeremy Everitts, James C. Bednarz

In the title of this article, the word “effect” should be “affect.” The correct title is: Does flooding affect the apparent survival and body condition of a ground foraging migrant passerine? The correct citation is: Reiley BM, Benson TJ, Everitts J, Bednarz JC (2017) Does flooding affect the apparent survival and body condition of a ground foraging migrant passerine? PLoS ONE 12(4): e0175179. https://doi.org/10.1371/journal.pone.0175179
